# Life based on phosphite: a genome-guided analysis of *Desulfotignum phosphitoxidans*

**DOI:** 10.1186/1471-2164-14-753

**Published:** 2013-11-02

**Authors:** Anja Poehlein, Rolf Daniel, Bernhard Schink, Diliana D Simeonova

**Affiliations:** Genomic and Applied Microbiology and Göttingen Genomics Laboratory, Georg-August University Göttingen, D-37077 Göttingen, Germany; Laboratory of Microbial Ecology, Department of Biology, University of Konstanz, D-78457 Konstanz, Germany

**Keywords:** *Desulfotignum phosphitoxidans*, Dissimilatory anaerobic phosphite oxidation, Genome sequencing

## Abstract

**Background:**

The Delta-Proteobacterium *Desulfotignum phosphitoxidans* is a type strain of the genus *Desulfotignum*, which comprises to date only three species together with *D. balticum* and *D. toluenicum*. *D. phosphitoxidans* oxidizes phosphite to phosphate as its only source of electrons, with either sulfate or CO_2_ as electron acceptor to gain its metabolic energy, which is of exclusive interest. Sequencing of the genome of this bacterium was undertaken to elucidate the genomic basis of this so far unique type of energy metabolism.

**Results:**

The genome contains 4,998,761 base pairs and 4646 genes of which 3609 were assigned to a function, and 1037 are without function prediction. Metabolic reconstruction revealed that most biosynthetic pathways of Gram negative, autotrophic sulfate reducers were present. Autotrophic CO_2_ assimilation proceeds through the Wood-Ljungdahl pathway. Additionally, we have found and confirmed the ability of the strain to couple phosphite oxidation to dissimilatory nitrate reduction to ammonia, which in itself is a new type of energy metabolism. Surprisingly, only two pathways for uptake, assimilation and utilization of inorganic and organic phosphonates were found in the genome. The unique for *D. phosphitoxidans* Ptx-Ptd cluster is involved in inorganic phosphite oxidation and an atypical C-P lyase-coding cluster (Phn) is involved in utilization of organophosphonates.

**Conclusions:**

We present the whole genome sequence of the first bacterium able to gain metabolic energy via phosphite oxidation. The data obtained provide initial information on the composition and architecture of the phosphite–utilizing and energy-transducing systems needed to live with phosphite as an unusual electron donor.

## Background

The phosphorus requirements of living cells are usually covered by phosphate and readily hydrolysable phosphate esters. In some cases as well as under phosphate starvation conditions, reduced organic and inorganic phosphorus compounds can be assimilated alternatively [1, 2]. Several aerobic and anaerobic bacteria are able to oxidize hypophosphite (+I) and phosphite (+III) to phosphate (+V), and to incorporate the latter into their biomass [3–9]. Phosphite can also be oxidized by *P. stutzeri* WM88 under denitrifying conditions when supplied as sole phosphorus source [7]. Whereas the phosphite oxidation pathways for assimilation purposes are well understood very little is known about the energetic side of this process. In addition to inorganic phosphonate (phosphite), a wide range of organo phosphonates, compounds bearing stable carbon-phosphorus (C-P) bonds, are known to be oxidized and degraded aerobically as P- and/or C-sources [1, 2, 10–12].

*Desulfotignum phosphitoxidans* is a rod-shaped Gram-negative bacterium that is able to grow with phosphite as a single electron donor and CO_2_ as the only carbon source. It grows slowly, with a doubling time of 72 to 80 h and is able to oxidize phosphite, fumarate, pyruvate, glycine, glutamate, maleate and other substrates with concomitant reduction of sulfate to sulfide. The strain can grow as a homoacetogen by reducing CO_2_ to acetate. In addition the strain is unable to utilize ethanol or lactate as substrate, which is unusual for a SRB [13]. Phylogenetically, *D. phosphitoxodans* is a member of the *Deltaproteobacteria*, order *Desulfobacterales*, *Desulfobacteriaceae* family, and is the first and unique bacterium to date that is able to utilize phosphite as electron donor in its energy metabolism.

The oxidation of phosphite to phosphate with sulfate as electron acceptor results in Δ*G*°'= -364 kJ per mol sulfate, or -91 kJ per mol phosphite. If CO_2_ is used as electron acceptor the Gibbs free energy of the reactions is ΔG°'= -308 kJ per mol acetate produced, or -77 kJ per mol phosphite oxidized, as previously described [13]. These energy spans allow for ATP yields of 1 to1.5 mol ATP per mol phosphite oxidized.

One major difference between previously described phosphite-assimilating bacteria and *D. phosphitoxidans* was found in the phosphite-oxidizing gene clusters, namely, the lack of an ABC-type phosphite uptake system and the presence of five new genes sharing no homology with any other gene known to participate in phosphite oxidation [14]. This finding together with the bacterium’s ability to use phosphite as electron donor in its energy metabolism opens a new field for exploration of a highly specific microbial lifestyle. The genome sequence and reconstructed metabolic pathways of *D. phosphitoxidans* presented here provide the first glimpse on the genetic properties of this strain. This work shows as well that the bacterium possesses distinct systems to handle phosphorus compounds – as energy and as unique P sources, which distinguishes it from all other Bacteria.

## Methods

### Media and growth conditions

*D. phosphitoxidans* strain FiPS-3 (DSM 13687) was grown anaerobically under a N_2_/CO_2_ (90:10, v/v) headspace, at 30°C in mineral medium, supplemented with 10 mM phosphite and 10 mM sulfate [15], or with 10 mM nitrate as the final electron acceptor. Multiple 1-liter cultures were used to obtain cells for DNA extractions and scanning electron micrographs.

### Genome sequencing

The genome sequencing strategy was described previously [16]. Briefly, *D. phosphitoxidans* genomic DNA was isolated with Purgene Core Kit B (Qiagen, Hilden, Germany) and MasterPure™ complete DNA purification kit (Epicentre, Madison, USA). Plasmid extractions from 4 separate cultures in quadruplicate were performed with the plasmid purification kit QIAGEN (QIAGEN, Hilden, Germany), and digested in single reactions with the restriction endonucleases HindIII, PstI, NdeI and MfeI (Thermo Fisher Scientific, Fermentas GmbH, Germany). The obtained fragments were separated on 1% agarose gels; the plasmid restriction map and its size were confirmed.

The extracted DNA was used in a combined sequencing approach using a 454 GS-FLX TitaniumXL system (Titanium GS70 chemistry, Roche Life Science, Mannheim, Germany) and the Genome Analyzer II (Illumina, San Diego, CA, USA). Shotgun libraries resulted in 13.76× coverage from 176.236 reads for 454 shotgun sequencing and 102.45x coverage from 7.344.206 of 112 bp paired-end Illumina reads, respectively. The initial hybrid *de novo* assembly employing MIRA software resulted in 149 contigs. PCR-based techniques and Sanger sequencing [17, 18] together with the Gap4 (v.4.11) software were used to close the gaps. The software used for automatic gene predictions were YACOP and GLIMMER [19], while RNAmmer and tRNAscan were used for identification of rRNA and tRNA genes, respectively [20, 21]. The functional annotation of the protein-coding genes was initially carried out with the IMG/ER (Intergrated Microbial Genomes/Expert Review) system [22] and manually curated by using the Swiss-Prot, TREMBL and InterPro databases [23].

The multiple sequence alignments were performed with ClustalW, with default settings [24].

### Electron microscopy

Cells were harvested by filtration, through a 0.2 μm PTFE filter and washed with 200 mM Na-cacodylate buffer. Cells were fixed in 200 mM Na-cacodylate buffer containing 180 mM glucose, 6% formaldehyde and 4% glutaraldegyde and 30, 50, 70 or 90% absolute ethanol on 5 nm thick gold-palladium grids.

## Results and discussion

### General features

**Figure 1:**
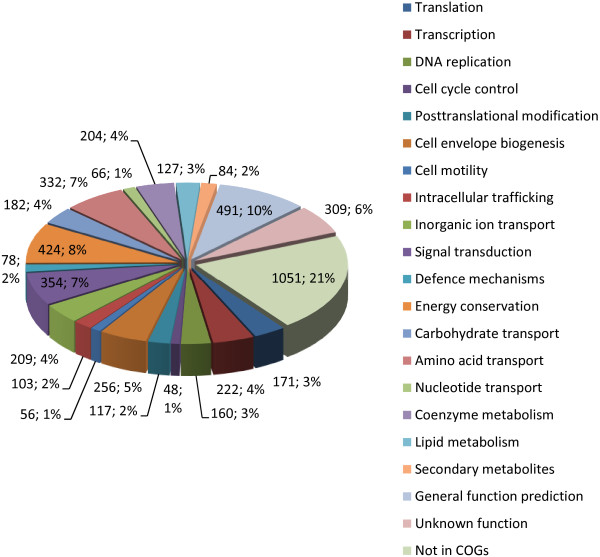
**Graphical representation of the functional groups clustering of** ***D. phosphitoxidans*** **genes.** Data is presented as the total gene count per group and in % of the total 4646 genes.

The genome of *D. phosphitoxidans* contains one circular chromosome (4,998,761 bp) and a small plasmid (7,714 bp). The average G+C content is 51.26%. Of the 4699 putative genes identified, 4646 coded for proteins of which 3609 (76.69%) were assigned to functions and 1037 (22.32%) had no database match (Tables 1 and 2, Figure 1). The genome contains two gene copies for each 5S, 23S rRNA and 16S rRNA, and 47 tRNAs.

**Table 1:** **General features of the** ***D. phosphitoxidans*** genome*

Category	Amount
DNA contigs/scaffolds	38
DNA total number of bases	4,998,761
Coding number of bases	4,534,179
G+C content	51.26%
ORFs^†^, total	4699
RNA genes	53 (1.13%)
rRNA genes	6 (0.13%)
tRNA genes	47 (1.00%)
Protein coding genes	4646 (98.87%)
With assigned functions	3609(76.80%)
Without function prediction	1037 (22.07%)
With COGs	3648 (77.63%)
With KOGs	1430 (30.43%)
With Pfam	3810 (81.80%)
With TIGRfam	1373 (29.22%)
In paralog clusters	3524 (74.99%)

*According to IMG database (https://img.jgi.doe.gov).

^†^ORFs, Open reading frames, coding regions.

**Table 2:** **Functional distribution of orthologous protein groups of** ***D. phosphitoxidans*** **genome (COG classification)**

COG categories	Gene count	% of total (4699)
**I. Information storage and processing:**		
Translation, ribosomal structure and biogenesis	171	3.63
Transcription	222	4.72
DNA replication, recombination and repair	160	3.40
**II. Cellular process:**		
Cell cycle control and division, chromosome partitioning	48	1.02
Posttranslational modification, protein turnover, chaperones	117	2.49
Cell envelope biogenesis, outer membrane	256	5.45
Cell motility	56	1.19
Intracellular trafficking, secretion, vesicular transport	103	2.19
Inorganic ion transport and metabolism	209	4.45
- Phosphorus metabolism (phosphate, phosphite and organophosphonates	31	
- Sulphur and nitrogen	22	
- Other	156	
Signal transduction mechanisms	354	7.53
Defence mechanisms/Stress	78	1.66
**III. Metabolism:**		
Energy production and conversion	424	9.02
Carbohydrate transport and metabolism	182	3.87
- Phosphorus – related (PTS system and sugar phosphate permeases)	14	
Amino acid transport and metabolism	332	7.06
Nucleotide transport and metabolism	66	1.41
Coenzyme metabolism	204	4.34
Lipid metabolism	127	2.70
Secondary metabolites biosynthesis, transport and catabolism	84	1.79
**IV. General function prediction only**	491	10.45
**V. Unknown function**	309	6.58
**VI. Not in COGs**	1051	22.37

The phage identification was done by using the ProphageFinder tool [25] and subsequent manual annotation of the genes. Within the suggested region, only 1 prophage (Dpo_5c01030-Dpo_5c01350) in the genome of *D. phosphitoxidans* was identified*.* Typical genes coding for phage proteins such as integrase, portal protein, tail tape measure protein, several capsid proteins as well as a phage terminase are located in this region. Immediately upstream of this region, a gene coding for Cro/C1-type HTH DNA-binding domain-containing protein is located (Dpo_5c00890). This protein is often associated with prophage regions, but its function remains unclear.

**Figure 2:**
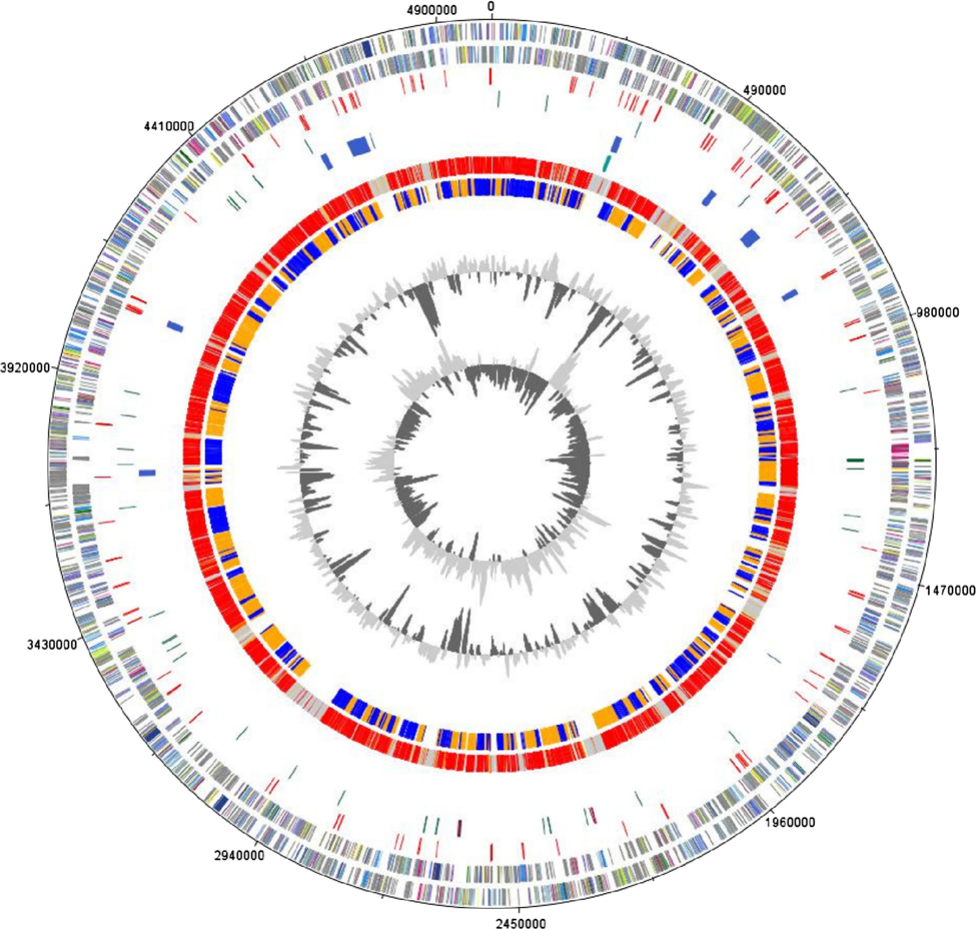
**Comparison of the chromosome of** ***D. phosphitoxidans*** **with** ***D. balticum*** **visualized with DNAPlotter.** From outside toward the centre of the figure: The red lines on the outer border show the gaps between the contigs. Genes encoded by the leading and the lagging strand are presented on circle 1 and 2, and marked in COG colours. Transposases (circle 3), rRNA-clusters and tRNAs (circle 4) are shown in red, pink and green. Genomic islands (circle 5) are shown in dark blue and were numbered clockwise, starting from 0. The *ptxEDptdFCGHI* operon is shown on circle 6 and marked in petroleum blue. A genome comparison based on bidirectional blasts were done against the genome of *D. balticum* (circle 7). The core genome is illustrated in red to light yellow and the pan genomes in grey with the following colour code: grey >e^-20^-1; light yellow <e^-50^- >e^-20^; gold <e^-50^->e^-90^ light orange: <e^-90^->e^-100^ orange: <e^-100^->e^-120^ red: <e^-120^-0. The cluster analysis resulting from the bidirectional blasts is shown in circle 8. Both inner circles represent the G+C content and the G+C skew of the chromosome.

With the help of the Semi-Automatic IS Annotation tool ISsaga [26] we could identify 120 transposable elements in the genome of *D. phosphitoxidans*, which were assigned to 20 different families. 48.25% of these genes can be allocated to unclassified IS families (ISNC), whereas the most abundant family within these group is the ISNCY_ssgr_ISDol1 family where 42 transposable elements could be assigned to. More than 15% of the transposable elements found in the genome were classified as members of the ISL3 family and more than 7% could be dedicated to the IS91 group. Transposable elements are often markers for genome plasticity and horizontal gene transfer, which are indicated by genomic islands (GI). These regions usually differ in G+C content and Codon Adaptation Index (CAI) from the average of the genome [27, 28]. By using the IslandPath-DIMOB software of the Islandviewer-platform we could identify 10 genomic islands with an overall size of 151 kb making up only 3% of the entire genome and their sizes range between 4 kb and 42 kb. Most of the genes located within the genomic islands are coding for hypothetical proteins, transcriptional regulators, transposases and recombinases. In the largest genomic island (GI 9, Figure 2[29]) 40 protein-encoding genes are located. Most of them are hypothetical, but an operon is coding for a complete type I restriction modification system (Dpo_8c00870-Dpo_8c00890) is also present. During annotation and analysis of the *D. phosphitoxidans* genome we have identified five additional type I, two type II and two type III restriction modification systems. One genomic island (GI 1, Figure 2) is very interesting and extremely important for the metabolism of *D. phosphitoxidans* since the *ptxEDptdFCGHI* operon (Dpo_11c01210-Dpo_11c01260) coding the proteins involved in phosphite uptake and oxidation, as well as the transcriptional regulator that probably regulates this gene cluster, are located on it. We report on this feature in more details in a later chapter concerning the metabolism of inorganic reduced P compounds.

Two operons coding for Cas proteins involved in the prokaryotic CRISPR/cas defense system could be identified in the genome of *D. phosphitoxidans*. The first operon (Dpo_3c03500-Dpo_3c0350) consists of two metal-dependent nucleases (Cas1 and Cas2) representing the universal *cas* genes, several genes code for proteins of the RAMP (Repeat-Associated Mysterious Proteins) superfamily (*csm2-5, cas6*) which are characteristic for type III CRISPR-Cas systems. The presence of *cas10* a gene coding for an HD-hydrolase allows further classification as the III-A/MTUBE subtype. The second operon (Dpo_7c00020-Dpo_7c00090) also consists of *cas1* and *cas2*, in addition a gene coding for a RecB family exonuclease (*cas4*) and Cas5 and Cas7 are present in this operon. According to the polythetic classification this cluster can be allocated to the I-C/DVULG subtype due to the presence of Cas3 and Cas8c the signature proteins for this subtype [30]. The presence of ten different restriction modification systems and two operons coding for a CRISPR/cas defense mechanism might be an explanation that there is only one prophage existing in the genome of *D. phosphitoxidans.*

### Comparison with other genomes

The *D. phosphitoxidans* genome falls within the 90% of the most frequently occurring bacterial genome sizes in the range of 4.5 to 5.5 Mbp. When the 4646 protein coding ORFs of *D. phosphitoxidans* were compared pair-wise to other complete individual bacterial genomes, belonging to bacterial Phyla Firmicutes, Chlorobia, Spirochaetes and Proteobacteria, the best BLAST hits revealed closest associations to members of the *Deltaproteobacteria*, order *Desulfobacterales*, belonging to the family *Desulfobacteraceae*. The *Desulfobacteraceae* is a highly versatile family containing 23 distinct genera and more than 42 species, as shown on the List of prokaryotic names webpage (http://www.bacterio.net/classifgenerafamilies.html, search done on 30^-th^ of July 2013). The highest numbers of best BLAST hits 3400 ORFs (73.18%) and 2355 ORFs (50.68%) were found against the genome of *Desulfotignum balticum* DSM 7044 and *Desulfobacterium autotrophicum* strain HRM2. However, the operon coding the phosphite oxidation ability in *D. phosphitoxidans* is unique for this strain and shows that it has been acquired through a lateral gene transfer from another yet unknown bacterium (Figure 2, circle 6). When compared to the genomes of *D. balticum* and *D. autotrophicum*, the genome of *D. phosphitoxidans* takes a middle place - it has one plasmid similar to *D. autotrophicum*, but the number of rRNA genes and the overall size of the genome is closest to those of *D. balticum*.

A comparison of the *D. phosphitoxidans* genome with the genomes of all sequenced organisms in the IMG database revealed that potential genes derived from Archaea or Eukaryotes were not present [31].

### Substrate tests and metabolic pathways constructions

The information obtained from the sequenced genome on substrate degradation and different electron acceptors was confirmed through growth experiments and compared with previously published data [13, 14]. In addition to prior known growth substrates, we observed growth with carbon monoxide and maleate. No growth was observed with benzoate or 4-hydroxybenzoate. For both degradation pathways only the gene encoding the central enzyme 4-hydroxybenzoyl-CoA reductase (EC 1.3.7.9) is missing. Further, we found that the strain grows autotrophically with phosphite as electron donor and nitrate as electron acceptor.

### Central metabolism

*D. phosphitoxidans* can grow heterotrophically with glucose, lactose, or galactose as carbon source. Hexoses are probably imported through a PTS-system (EC 2.7.1.69) to form glucose-6-phosphate; the further path leads via fructose-6-phosphate into glycolysis to form phosphoenolpyruvate and pyruvate. Surprisingly, the fructose-1,6-bisphosphate aldolase (EC 4.1.2.13) is missing in the genome. The function of this enzyme could be replaced by reactions of the pentose phosphate cycle: Two subsequent transaldolase reactions could convert fructose-6-phosphate with erythrose-4-phosphate (EC 2.2.1.2, Dpo_2c03050) to glyceraldehyde-3-phosphate and sedoheptulose-7-phosphate which recycles erythrose-4-phosphate with production of a further glyceraldehyde-3-phosphate through a transaldolase reaction. The pentose phosphate cycle is complete and could allow utilization of pentoses. Growth with arabinose and xylose has been documented before [13], but could not be reproduced in recent experiments. The incomplete glycolytic pathway could explain why growth of this bacterium with sugars is rather slow and poor. This is consistent with the fact that most sulfate-reducing bacteria do not use sugars at all.

**Figure 3:**
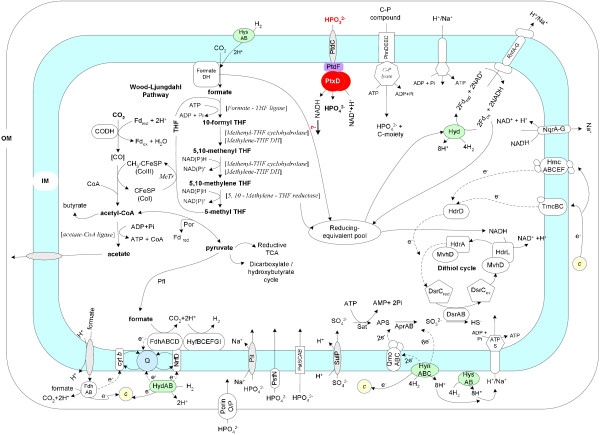
**Pathways of dissimilatory electron flow, autotrophic CO**_**2**_ **assimilation and phosphorus metabolism in** ***D. phosphitoxidans*** **.** Arrows indicate established metabolic paths; dashed arrows indicate hypothetical electron flows. For the sake of simplicity, oxidation pathways for organic substrates are not shown. Phosphonate dehydrogenase is shown in red; the NAD(P)-dependent epimerase/dehydratase PtdF is shown in purple; high-affinity symporters are shown in grey; hydrogenases are shown in green and the soluble cytochromes are shown in yellow.

For autotrophic growth, *D. phosphitoxidans* uses the carbon monoxide (CO) dehydrogenase (Wood-Ljungdahl) pathway for which all necessary enzymes were found in the genome (Figure 3). In addition, CO dehydrogenase activity had been documented previously [13]. This pathway is also used in lithotrophic growth with phosphite as electron donor and CO_2_ as sole electron acceptor, to form acetate as reduced reaction product through homoacetogenesis. All enzymes of the citric acid cycle are present, except for succinate dehydrogenase. Presence of fumarate reductase (menaquinone) instead indicates that the C_4_ –dicarboxylic acid branch of the citric acid cycle is used in the reductive direction. Therefore the reactions of the citric acid cycle in this bacterium serve merely as a supply system for biosynthesis of amino acids and other important building blocks. Presence of genes encoding citrate synthase (EC 2.3.3.1, Dpo_14c00120, Dpo_20c00240) and absence of potential genes for ATP-citrate lyase (EC 2.3.3.8) indicates that the reductive citric acid cycle is not used for autotrophic CO_2_ assimilation in this bacterium. In addition putative genes encoding key enzymes of the glyoxylate shunt are missing.

In gluconeogenesis during autotrophic growth, pyruvate is formed reductively from acetyl-CoA through pyruvate synthase (EC 1.2.7.1, Dpo_10c00680 - Dpo_10c00700) and can be activated to phosphoenolpyruvate through pyruvate phosphate dikinase (EC 2.7.9.1, Dpo_5c02610; Dpo_6c01290) or pyruvate water dikinase (EC 2.7.9.2, Dpo_2c00560; Dpo_3c01990; Dpo_3c02000; Dpo_9c01030). Gluconeogenesis is driven by employing steps of the pentose phosphate cycle to produce hexose units for synthesis of cell wall polysaccharides. This synthesis starts from fructose-6-phosphate via glutamine: fructose-6-phosphate transaminase (EC 2.6.1.16, Dpo_2c00930) to glucosamine-6-phosphate; all genes necessary for synthesis of peptidoglycan via acetylglucosamine and addition of the lactyl ether to the biosynthesis of the amino acid side branch were found to be present.

In the pathway for tetrahydrofolate biosynthesis, all required genes despite the dihydrofolate reductase gene were found in the genome, but a thymidylate synthase gene was identified which has been described in several other genomes as a bifunctional thymidylate synthase/dihydrofolate reductase enzyme. The adenosylcobalamin (vitamin B_12_) biosynthesis pathway is almost complete, where all genes for synthesis of precorrin-3A were found. Of the two pathways for cobalt insertion, i.e., the “early” and the “late” cobalt insertion pathway, some enzymes are missing in either case. The “early” cobalt insertion lacks the precorrin-2 cobalt chelatase and the cobalt-precorrin-7 (C15)-methyltransferase genes, while the “late” one has no precorrin-3B synthase, precorrin-6B synthase, and cobaltochelatase genes. These functions are covered by alternative enzymes [32].

Synthesis of coenzyme A is complete, as is the synthesis of ferredoxins for transfer of low-potential electrons, e.g., for reduction of CO_2_ to CO.

### Metabolism of inorganic phosphate (Pi)

The genome of *D. phosphitoxidans* contains the genes for both two-component signal transduction pathways of the Pho regulon, encoded by *phoBR* (Gram negative bacteria), and *phoPR* (Gram-positive bacteria), such as *Clostridium acetobutylicum* or *Bacillus subtilis*. This is another unique feature of the *D. phosphitoxidans* genome in comparison to other sulfate-reducing bacteria (SRB). Of 95 sequenced sulfate-reducers genomes available from IMG database, phoBR system was annotated in most of them, including *D. balticum*, *D. autotrophicum* HRM2, *D. vulgaris vulgaris* Hildenborough, whereas the *phoPR* system was coded in only 6 genomes including one Gram-positive representative - *Desulfosporosinus* sp. Strain OT. Unexpectedly, we could not identify a gene coding for a periplasmic alkaline phosphatase *phoA* in the genome. It was shown in *E. coli* that this enzyme is involved in the utilization of phosphite as phosphorus source under phosphorus starvation [8] and plays also a major role in the supplementation of phosphorus during growth under phosphorus limitation and/or uptake of phosphate esters. PhoA is not present as well in the *D. autotrophicum* HRM2 genome, but in most of SRB there is at least one copy of a periplasmic alkaline phosphatase gene. In the genome of the closest relative of *D. phosphitoxidans*, *D. balticum,* we found a gene product which belongs to the alkaline phosphatase superfamily but was not unequivocally annotated as an alkaline phosphatase. In addition, we could not identify unequivocally a phosphodiesterase (PhoD) gene ortholog, but nevertheless, several genes coding for phosphoric mono- and di-ester hydrolases and two copies (Dpo_7c02380; Dpo_1c03120) of the inorganic diphosphatase (Ppa) were present in the genome. The diphosphate phosphohydrolase is known to act on phosphoric acid anhydrides with a broad specificity that varies according to the source and the activating metal ion, thus being identical with the alkaline phosphatase (EC 3.1.3.1) or the glucose-6-phosphatase (EC 3.1.3.9) [33, 34].

The phosphate requirements of *D. phosphitoxidans* can be covered via all three Pi uptake systems known [35]: i) a low affinity system (Pit); ii) a high affinity (Pst) system, and iii) a phosphate ester uptake system PTS (fructose and mannose specific). Although the strain grew with glucose, neither the genes encoding a Pi-linked antiport of glucose-6-P (UhpT, UgpA or UgpB), nor the GlpT protein of the antiport system for the transport of *sn*-glycerol-3-P were identified. Nevertheless, 7 genes of the PTS system (including *ptsI* Dpo_5c02530, *ptsH* Dpo_5c02520 and *ptsN* Dpo_3c02590) and 7 genes coding for sugar phosphate permeases were identified in the genome (Figure 3, Table 2), which is in agreement with a previously reported ability of the strain to grow slowly with C6 and C5 sugars [13]. The low-affinity (Pit) system is represented by two Na^+^/phosphate symporter genes (Dpo_7c01660; Dpo_4c03310) and one phosphate-selective porin (Dpo_3c01140). A comparison between *D. phosphotoxidans* genome and the other SRB genomes shows that only *D. autotrophicum* HRM2 and *D. toluolica* Tol2 genomes contained one copy of Pit each. Surprisingly none of the publicly available SRB genomes contained a gene orthologous to the phosphate-selective porin found in *D. phosphitoxidans*. The Pi starvation–inducible high-affinity Pst system is represented in *D. phosphitoxidans* by only one complete set of genes organized in an operon structure (*pstSCAB,phoU*), whereas *pstS*, *pstA* and *pstB* genes were found in multiple copies (Figure 3, Table 2). Also, this system is the most abundant one amongst SRB, where all strains sequenced to date possess it in at least one copy.

### Metabolism of organic reduced P compounds (organophosphonates)

**Figure 4:**
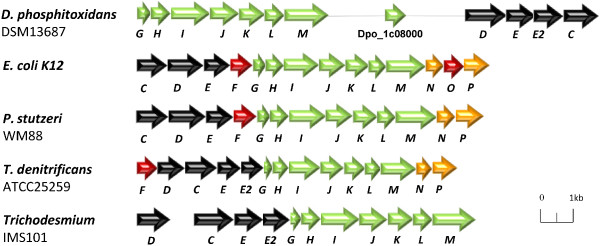
**Comparison of the** ***phn*** **gene clusters of** ***D. phosphitoxidans*** **with other bacteria.** The Phn (C-P lyase) complex allows the uptake and growth on a broad spectrum of organophosphonates. The genes involved in phosphonate transport are shown in black, C-P lyase catalytic subunits in green, regulation in red and the accessory genes in yellow.

In addition to the Pi supplementation systems, *D. phosphitoxidans* is able to cover its phosphorus requirements via reduced organic phosphorus compounds. Of the four distinct organophosphonate degradation pathways identified so far, only the carbon-phosphorus lyase (*phn*) gene cluster was found in *D. phosphitoxidans*. The gene locus is split in two parts, *phnGHIJKLM* (the minimal region required for C-P bond cleavage, Dpo_1c08140 to Dpo_1c08080) and *phnDEEC* (phosphonic acid transporter region, Dpo_1c07910 to Dpo_1c07940), which are separated by 13 genes (Figure 4). It is shorter than the 14-gene C-P lyase locus of *E. coli* and misses the PhnNP accessory proteins and the PhnFO assumed to be involved in the regulation of the cellular phosphorus level [11, 36]. Nevertheless, *D. phosphitoxidans* utilizes alkylphosphonates (triethyl phosphite, diisopropyl phosphite), organophosphonates (2-aminoethyl phosphonate, tert-butylphosphonic acid) and phenylphosphonate as the only phosphorus source for growth, but not as carbon or energy source. This is in accordance with the previously shown broad substrate spectrum specificity of the C-P lyase pathway [12] in several Pseudomonads*, Escherichia hermannii, Bacillus megaterium, Actinomyces* spp*.* and the marine diazotroph *Trichodesmium*[10]. To compare, of 95 whole genome sequenced SRB in IMG data base, only 11 more contained a C-P lyase operon, excluding the closest relative *Desulfotignum balticum* DSM 7044 ( by August, 1^-st^,2013). The C-P lyase operon was found in the genomes of both Gram (-) and Gram (+) SRB.

The pathways of 2-aminoethyl phosphonate degradation require the presence of either a phosphonatase that cleaves 2-phosphonoacetaldehyde to acetaldehyde and phosphate or the phosphonoacetaldehyde dehydrogenase coupled to phosphonoacetate hydrolase (PhnWY-PhnA) pathways [37, 38]. Strikingly, no gene homologous to phosphonatase (PhnX, EC 3.11.1.1) has been found in *D. phosphitoxidans*. This is in consistence with the other SRB genomes available to date. In addition we found that 18 out of the 95 SRB strains had a *phnA* coding for phosphonoacetate hydrolase. An interesting observation is that the PhnA was found only in Gram (-) SRB, but not in Gram (+). In conclusion, only three SRB species contained genes coding for both organophosphonates degradation pathways, the C-P lyase and the phosphonatase pathway: *Desulfomicrobium baculatum* × DSM 4028, *Desulfobacter curvatus* DSM 3379, and *Desulfobacula toluolica* Tol2.

### Inorganic reduced P compounds metabolism

**Figure 5:**
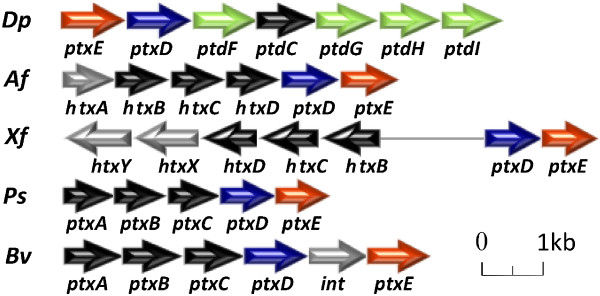
**Operons involved in phosphite oxidation.** *D. phosphitoxidans* (*Dp*), *A. faecalis* WM2072 (*Af*), *X. flavus* WM2814 (*Xf*), *P. stutzeri* WM88 (*Ps*) and *B. vietnamiensis* G4 (*Bv*): regulatory genes (red), phosphite oxidation (blue), phosphite uptake (black). The specific genes for *D. phosphitoxidans* phosphite oxidation gene cluster, without homologs in the other species, are shown in green. The *htx* genes (grey) are involved in hypophosphite uptake and oxidation.

The strain’s dual ability to utilize phosphite as a single phosphorus source and/or as an electron source to support its energy metabolism distinguishes it from all other phosphite oxidizers known to date. The gene cluster *ptxEDptdFCGHI* of *D. phosphitoxidans* is encoded on the chromosome. Already was shown before that the partial cluster *ptxD-prdFCG* conferred phosphite uptake and oxidation ability to *D. balticum* DSM 7044 as a host strain, but did not allow the use of phosphite as an electron donor for chemolithotrophic growth [14]. The *ptxEDptdFCGHI* operon forms a genomic island which is surrounded by two transposases. This suggests its lateral gene transferfrom another organism (Figure 2). The *D. phosphitoxidans* genome contained only one copy of a phosphonate dehydrogenase (*ptxD*) and, in contrast to the previously described phosphite oxidation gene clusters, the uptake of phosphite is not an ATP-dependent process. Phosphite uptake in this strain proceeds via a high-affinity MFS protein PtdC (Figure 3 and Figure 5), suggesting that its primary role is to supply an electron donor for the energy metabolism. To date, only in two other SRB genomes a gene was annotated which presumably codes for a phosphonate dehydrogenase (PtxD), but there are no physiological proofs of phosphite oxidation ability in those strains (search performed on 02. August 2013). The two strains are *Desulfovibrio putealis* DSM 16056 and *Thermodesulfovibrio thiophilus* DSM 17215.

Further, no other genes known to be involved in the assimilation of reduced P-compounds as for example the *htx* locus which takes part in the uptake and oxidation of hypophosphite (P+I) to phosphate in *P. stutzeri* WM88 [39] were identified in the genome of *D. phosphitoxidans*. The same is true for all other sulfate-reducers.

### Sulfur metabolism

As an obligate anaerobe and typical sulfate-reducer, *D. phosphitoxidans* is unable to use oxygen, but grows with sulfate, sulfite, thiosulfate, nitrate, CO_2_ or fumarate as electron acceptor. Sulfate reduction proceeds via ATP sulfurylase and APS reductase to sulfite, which is further reduced to H_2_S by dissimilatory sulfite reductase DsrAB (Figure 3). The genome contains in addition one gene and one candidate gene for polysulfide reductase NrfD (Dpo_6c00850; Dpo_1c08450) that transfers electrons from the quinone pool, and for a thiosulfate reductase PhsAB (Dpo_3c03810; Dpo_3c03820). In addition, a quinone-coupled membrane-bound QmoABC complex is present in the genome, which presumably is involved in the direct transfer of electrons to the APS reductase (AprAB) as shown in Figure 3[40]. Another complex related to the heterodisufide reductase and involved in menaquinol oxidation in sulfate reducers, HmeABCDE, described for the first time in *Archaeoglobus fulgidus*, is present as well in the genome of *D. phosphitoxidans* (Dpo_2c02400, Dpo_7c01170 to Dpo_7c01210) [41]. The same complex few years later was isolated and described in *Desulfovibrio desulfuricans* ATCC 27774 by another group, and was named DsrMKJOP [42, 43]. Interesting point is that the subunits of this complex are coded in two different loci on the chromosome of *D.phosphitoxidans*, and apart from any other genes and complexes involved in sulfate reduction. This is an atypical constellation in comparison with the majority of the sulfate reducing organisms [43]. Two membrane-bound redox complexes, the HmcABCDEF having a function analogous to the Hme complex and a TmcABCD complex (a simplified version of the Hmc complex [44]), are present in a single copy each. The DsrC gene is present in two copies (Dpo_2c03440; Dpo_5c02880) and thus can form a functional unit with DsrAB.

### Nitrogen metabolism

Nitrate is reduced to nitrite via a respiratory nitrate reductase type I NarGHI, complemented with a gene coding for NapC (Dpo_10c01280), the electron-transferring cytochrome *c* subunit of the periplasmic nitrate reductase NapABCDE [45]. In addition, the genome contains a cytoplasmic nitrate reductase NasAB in a single copy. Nitrite is further reduced through a NAD(P)-dependent dissimilatory nitrite reductase (EC 1.7.1.4, Dpo_11c00510) or a cytochrome c nitrite reductase (EC 1.7.2.2, Dpo_4c02890) to ammonia. A respiratory (NO-forming) nitrite reductase (EC 1.7.2.1) was not present, whereas the successive nitric oxide reductase NorB was (Dpo_19c00290). Further, nitric oxide reduction or nitrite reduction to ammonia can proceed as well through ammonia:ferricytochrome-c oxidoreductase (EC 1.7.2.2) which is known to act on NO and hydroxylamine as substrates [46]. Finally, the inspection of the genome reveals 6 genes sharing homology with the subunits of the ATP-hydrolysing dinitrogen oxidoreductase (EC 1.19.6.1). The ability of the strain to use nitrate as an electron acceptor was proven by growth tests with 10 mM phosphite plus 15 mM nitrate, in which a maximum of 2.25 mM NH_3_ was accumulated in the medium. This corresponds to the expected ammonia formation (2.5 mM) if coupled stoichiometrically to the oxidation of 10 mM phosphite.

### Energy conservation and electron flow

The *D. phosphitoxidans* genome contains all genes necessary for the Wood-Ljungdahl pathway, which operates both in CO_2_ assimilation and energy metabolism (Figure 3). The genome lacks homologues of acetate kinase and phosphate acetyltransferase, although the latter activity [47, 48] but not acetate kinase activity was measured in cell-free extracts of cells grown autotrophically with phosphite. Moreover, four genes coding for putative acetyl/butyryl CoA transferases and three genes coding for acetyl-CoA synthetase (ADP-forming) were found; these enzymes are used by acetate-forming *Archaea* to synthesize ATP during acetate formation [49], and could take over the role of acetate kinase in *D. phosphitoxidans*.

Two soluble formate dehydrogenases, the FdhAB (Dpo_2c03450; Dpo_2c03460) and the NAD-dependent formate dehydrogenase I (EC 1.2.1.2) clusters, are present in the genome. Both enzyme activities were measured in extracts of autotrophically grown cells [50, 51]. In addition to the aforementioned formate dehydrogenases, a membrane-associated FdhABD formate dehydrogenase was found. These findings suggest energy conservation by a formate cycle, as shown in Figure 3.

Amongst all 95 genomes of sulfate-reducing bacteria, only three contain NuoEFG genes from the 14-subunits NADH:ubiquinone oxidoreductase complex. The *Desulfovibrio magneticus* RS-1 and *Syntrophobacter fumaroxidans* MPOB harbour the entire NuoABCDEFGHIJKLMN cluster [42, 52], whereas *D. phosphitoxidans* contains only the EFG subunits. The genome of *D. phosphitoxidans* differs in the number of copies as well - four copies of the NuoEFG subunits forming the NADH dehydrogenase module of the NADH: ubiquinone reductase (H^+^-translocating) complex (EC 1.6.5.3), but no further *nuo* genes [44]. In *D. phosphitoxidans* genome, the NuoEFG subunits are organized in putative transcriptional units together with a CoB--CoM heterodisulfide reductase subunit HdrA, a methyl-viologen-reducing hydrogenase MvhD (*nuoEFGmvhD*; *nuoGEFmvhDhdrA*), formate DH (*nuoEFfdhF*), and electron transfer flavoproteins EtfAB (*hdrAmvhDnuoEFGdfhDetfAB*). This indicates that the NADH generated during phosphite oxidation can be the actual electron donor for intracellular reduction of ferredoxin.

The genome of *D. phosphitoxidans* contains a soluble succinate dehydrogenase,encoded in a single copy by *frdABC* (Dpo_4c03460 to Dpo_4c03480), and a candidate gene for FrdD transmembrane subunit (Dpo_4c03580) between a fumarase FumA subunit and the succinate-CoA ligase (ADP-forming) gene cluster. The lack of complex II activity was proven by enzyme assays in cell extracts [53].

**Figure 6:**
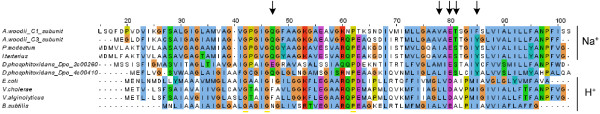
**Comparison of the** ***D. phosphitoxidans*** **’ two gamma subunits of the F**_**1**_ **F**_**0**_ **ATPases with the gamma subunits of Na** ^**+**^ **, and H** ^**+**^ **-translocating ATPases.** NCBI protein database accession numbers used: *Escherichia coli* (P68699), *Vibrio cholerae* (AAF95908), *Vibrio alginolyticus* (POA308), *Bacillus subtilis* (P37815), *Acetobacterium woodii*, C1 subunit (AFA47025.1) and C3 subunit (AFA47026.1), *Propionigenium modestum* (P21905), *Ilyobacter tartaricus* (Q8KRV3), *D. phosphitoxidans* (Dpo_2c00260), and (Dpo_4c00410). Arrow marks show the positions of the conserved amino acids for sodium ion binding. *D. phosphitoxidans* gamma subunit Dpo_2c00260 shows a non-conserved substitution at position 47 (glutamic acid instead of a glutamine), and a substitution at position 78 (methionine), whereas the Dpo_4c00410, differs in one amino acid, at position 78 (leucine), which is conserved amongst H^+^-translocating ATPases.

Unexpected for a strict anaerobe, the genome contains as well a gene coding for the CyoE subunit of the terminal cytochrome *bo* oxidase complex and homologs of the CydAB, the terminal cytochrome *bd* quinol oxidase subunits (Dpo_2c00830; Dpo_2c00840). Furthermore, four putative genes encoding subunits of the complex IV, cytochrome *c* oxidase (EC 1.9.3.1) were found (Dpo_12c00840 to Dpo_12c00870). Their presence might be related to an enhanced resistance towards nitrite rather than to aerobic respiration [54, 55]. The bacterial F_0_F_1_-type ATPase is present in two complete copies (Dpo_2c00210 to Dpo_2c00290), one of which is split into two clusters, one coding for the F_1_ unit (containing the αβγδϵ subunits) and another one coding for the F_0_ unit (*abc*-subunits). Surprisingly, an alignment of the two gamma subunits revealed closest relation to the Na^+^-dependent ATPases, rather than to the H^+^ dependent ones, as shown on Figure 6.

The electron flow during phosphite oxidation is still unknown, but the genome of *D. phosphitoxidans* reveals several options for respiratory electron transport through FMNH_2_-, FADH_2_- and NAD(P)H-linked reactions. Within the genome, the highest number of genes assigned to function (424 genes) is related to the energy conversion COG category, with exception of the general-function-prediction COG group (491 genes; Table 2, Figure 1). The number of *b*-type cytochromes in the genome is limited to three genes (Dpo_7c01210; Dpo_2c00850; Dpo_19c00130). Substantially higher is the number of putative *c*-type cytochromes - 20, including one tetra-heme cytochrome *c*_*3*_, three genes for cytochrome *c*_*552*_, and one for cytochrome *c*_*554*_. Furthermore, two putative genes for electron transport proteins, seven gene clusters *etfAB* (electron transport flavoproteins), and ten flavoproteins seven of which are flavodoxins were found in the genome. Fourteen Fe-S, two 2Fe-2S and 29 genes coding for 4Fe-4S-containing proteins indicate an electron transfer that couples substrate metabolism with redox carriers such as ferredoxins and/or NAD(P)^+^ in the cytoplasm of the cell. The soluble electron transfer machinery of the strain consists of a [NiFeSe]-periplasmic hydrogenase HysAB, a [NiFe]-membrane bound periplasmic hydrogenase HynCAB (EC 1.12.99.6), a HypABEDFC hydrogenase, and a soluble periplasmic [NiFe]-hydrogen: quinone oxidoreductase HydAB (EC 1.12.5.1). In addition, we found a six-gene cluster homologous to the ten-subunit Hyf complex encoding a membrane-associated H_2_-evolving respiratory [NiFe]-hydrogenase originally described for *E. coli*[56]. The genome contains a set of five genes homologous to the CoB-CoM heterodisulfide reductase (HdrABCDE) and ten copies of the MvhD methyl-viologen reducing hydrogenase. Together with the presence of several periplasmic hydrogenases, the HmcABCDEF and the TmcACBD complexes known to be involved in electron transfer from the periplasm to the cytoplasm during H_2_ oxidation [57], these findings indicate the ability of *D. phosphitoxidans* to use hydrogen in addition to formate as electron donor.

### Ion translocation and ATP synthesis

**Figure 7:**
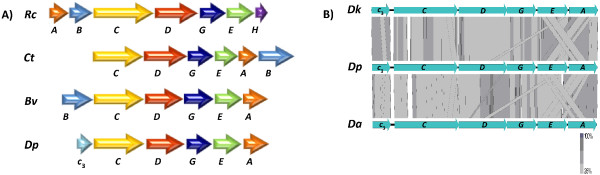
**Rnf clusters comparisons. A)** the *rnf*-type gene cluster in *D. phosphitoxidans* (*Dp*) and the *rnf* clusters of *R. capsulatus* (*Rc*), *C. tetani* (*Ct*) and *B. vulgatus* (*Bv*). Genes are aligned 5′ to 3′ and the identically colored genes are homologous. **B)** A tBlastx comparison of Rnf clusters from *Desulfotomaculum kuznetzovii* (*Dk*), *D. phosphitoxidans* (*Dp*) and *Desulfobacterium autotrophicum* HRM2 (*Da*). The graphical presentation was generated with Easyfig software, and a cut-off value of 1e-10 was used.

In the genome of *D. phosphitoxidans* a set of six genes (Dpo_1c03590 – Dpo_1c03640) was found of which five were identified as *rnfCDGEA,* those are coding subunits of the Rnf ion translocation system found in *Rhodobacter capsulatus*, *Clostridium tetani*, *Bacteroides vulgatus* and others [58–62] (Figure 7). The remaining gene (Dpo_1c03590) is a potential candidate to also belong to the Rnf complex, as it is a putative cytochrome *c*, containing a Fe-S center. The RnfABCDGEH complex of *R. capsulatus* is involved in electron transport to nitrogenase, driving the reversed electron transport from NADH to ferredoxin [59], whereas the gene clusters in Clostridia and *Bacteroides vulgatus* are believed to function in the generation of transmembrane ion gradients [58]. The presence of an Rnf-type complex in the genome of *D. phosphitoxidans* indicates that it might be involved in electron transport and energy conservation during phosphite oxidation. Our search in the IMG database against the RnfC, RnfD and RnfE subunits revealed 36, 19, and 11 SRB strains, which harboured them, respectively. However, only 9 SRB genomes contained an Rnf complex belonging to two of the four Rnf-complex types, shown in Figure 7. *D. autotrophicum* HRM2, *D. phosphitoxidans* and *Desulfotomaculum kuznetzovii* formed the type four Rnf complex in Figure 7A, presented in details in Figure 7B; *Db. postgatei* 2ac9, *D. toluolica* Tol2, *Desulfomonile tjedjei* DCB-1 DSM 6799, *Desulfosarcina* sp BuS5, *Desulfobacter curvatus* DSM 3379 and *Desulfovibrio aminophilus* DSM 12254 belong to the type two, and identical to the *C. tetani* Rnf complex, as shown in Figure 7A.

In addition to the Rnf complex, a Na^+^-dependent NADH:ubiquinone oxidoreductase NqrABCDEF complex was identified (Dpo_16c00300 to Dpo_16c00350), which is known to be typical of the respiratory chains of various marine and halophilic bacteria. Interestingly only 9 SRB genomes contained loci coding an Nqr complex, two of which are freshwater isolates - *Syntrophobacter fumaroxidans* MPOB and *Desulfococcus oleovorans* HxD3. The Nqr complex of *D. phosphitoxidans* is identical with the one found in *Vibrio alginolyticus*, *Vibrio cholerae,* and other marine and Gram-negative pathogens [63]. The primary function of this complex is the oxidation of NADH coupled to Na^+^ ion transport across the cytoplasmic membrane [60]. Surprisingly *D. balticum*, being the closest relative of *D. phosphitoxidans* and a marine bacterium, does not harbour either an Rnf, or an Nqr complex, although there are genes annotated as subunits of these complexes, below the trusted cut-off.

### Stress

The genome of *D. phosphitoxidans* contains genes for oxidative stress protection including one catalase-peroxidase (EC 1.11.1.21), three peroxiredoxins, six thioredoxins, one Fe- and one Ni- containing superoxide dismutase. This is in consistence with the other genome-sequenced SRB, 22 of them have as well a catalase-peroxidase gene. Seventy-five have at least one gene copy for peroxiredoxin and 61 of them harbour a superoxide dismutase gene (EC 1.15.1.1) as *D. phosphitoxidans* does. Surprisingly, only *D. phosphitoxidans* and *D. autotrophicum* had PrxU coding genes for selenocystein-containing peroxiredoxin. In addition, two genes of the superoxide reductase system (desulfoferrodoxin) as found in obligate anaerobes were identified in the genome of *D. phosphitoxidans*.

Several genes coding for efflux pumps have been identified that confer resistance against heavy metals and metalloids, including the arsenite efflux pump (ACR3). Both TolC-dependent multidrug resistance systems were found in the genome: the ABC-type, the MtdABC-TolC type and the AcrAB-TolC type, including some small RND-type multidrug resistance proteins and a macrolide-binding efflux protein. Similarly only 30% (or 28) of all SRB with sequenced genomes contained a Type I secretion outer membrane protein TolC including *D. balticum*.

Genes for heat/cold-shock proteins include five chaperones, *dnaK*, *dnaJ*, *grpE*, a small heat-shock protein Hsp20, and an Hsp70 protein, as well as two cold-shock proteins. Also 16 genes are present encoding the universal stress protein UspA, two solvent and several acid-stress response proteins.

### Regulation and signal transduction

The core polymerase (RpoA, RpoB, RpoC, RpoZ) along with four sigma factors σ-^70^ (RpoD), σ-^54^ (RpoN), σ-^32^ (RpoH) and σ-^24^ (RpoE) confer promoter specificity in the genome of *D. phosphitoxidans*. Strikingly, no gene coding for stationary phase sigma factor σ-^38^ (RpoS) or a flagella biosynthesis sigma σ-^28^ (FliA) were found. The genome of *D. phosphitoxidans* contains twenty-one σ-^54^-dependent transcriptional regulators, seventeen HTH-type transcriptional regulators and eighty-five response regulators out of 354 genes involved in signal transduction mechanisms. Thus, this functional group is the second biggest COG group identified with 8.86% in the entire genome (Table 2).

### Motility and taxis

**Figure 8:**
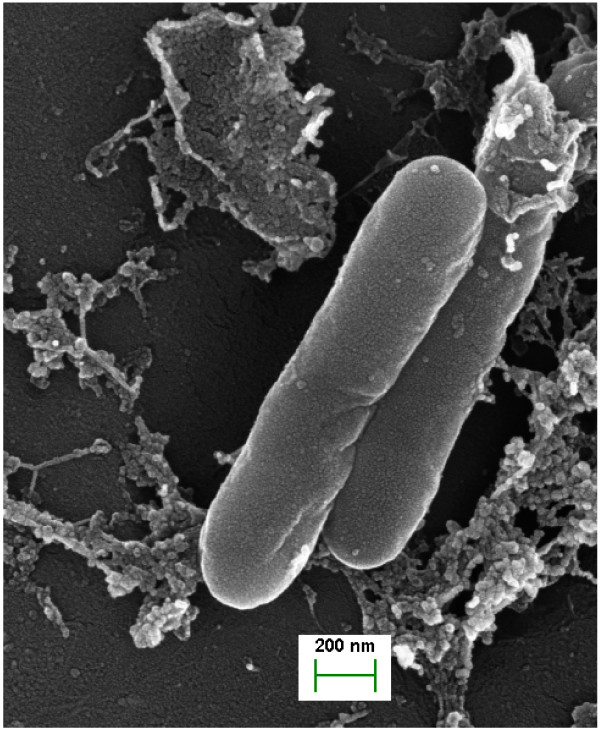
**Scanning electron micrograph of** ***D. phosphitoxidans*** **cells in the mid-exponential growth-phase.**

*D. phosphitoxidans* is a non-motile bacterium *sensu stricto* that lacks flagellar structural proteins, σ28 and anti- σ28 factor (FlgM). Also the *E. coli*-type master switch proteins FlhCD are absent (Figure 8). Instead, the bacterium appears to be motile via gas vesicles, for which one gene was unequivocally identified in the genome (Dpo_1c07060). In addition six methyl-accepting chemotaxis proteins and three methylases for methyl-accepting chemotaxis proteins were identified. Ten out of eleven MCP sensory proteins are involved in as yet unknown attractants and/or repellents for signal transduction via one CheA (Dpo_12c00440), two CheW (Dpo_1c03230, Dpo_12c00460) and two CheY-like proteins (Dpo_2c03950, Dpo_5c00010). Quorum signalling genes *luxOQ* (Dpo_1c07250, Dpo_10c00270) were specific only for *D. phosphitoxidans* in comparison to the 95 SRB genomes. *D. balticum* harbours only a *luxQ*. In addition, the *D. phosphitoxidans* genome contains the full set of conserved subunit genes involved in the synthesis of Type IV pili (*pilBCDQ*), except for the *pilA*.

## Conclusions

The whole genome sequence of *D. phosphitoxidans* has provided an opportunity to understand the nature of phosphite oxidation and thereby derived energy as a unique feature of this bacterium amongst all known bacterial species.

The *ptxEDptdFCGHI* locus involved in phosphite oxidation is a genomic island on the chromosome of the strain, flanked downstream by a putative transposase belonging to the resolvase/recombinase family and upstream with a rolling-cycle transposase. Also, this clearly indicates that the locus was subject to a lateral gene transfer from another organism, which suggests that this type of metabolism might have a broader distribution in different environments and subsequently an impact on the phosphorus cycle in nature.

Further, the presence of NuoEFG coding genes, forming the NADH dehydrogenase module of the NADH: ubiquinone reductase (H^+^-translocating) complex is as well unique for the genome of *D. phosphitoxidans* amongst all Gram (-) sulfate-reducing bacteria, and we expect that it plays a role in the energy conserving system.

Since phosphite can play an important role in the energetic metabolism of this bacterium, the strain has acquired additional both phosphorus two-component regulation systems - the PhoBR and PhoPR.

Finally, the narrow range of substrates that can serve as electron donors for this strain, its inability to grow via fermentation, and the surprisingly poor phosphonate uptake and utilization abilities, combined with the unique phosphite oxidation and energy conservation system of this strain, clearly underlines a highly specific and unique live strategy.
